# Nonspiking Interneurons in the *Drosophila* Antennal Lobe Exhibit Spatially Restricted Activity

**DOI:** 10.1523/ENEURO.0109-22.2022

**Published:** 2023-01-24

**Authors:** Jonathan E. Schenk, Quentin Gaudry

**Affiliations:** Department of Biology, University of Maryland, College Park, MD 20742

**Keywords:** *Drosophila*, interneurons, nonspiking, olfaction, sodium channel

## Abstract

Inhibitory interneurons are important for neuronal circuit function. They regulate sensory inputs and enhance output discriminability (Olsen and Wilson, 2008; Root et al., 2008; Olsen et al., 2010). Often, the identities of interneurons can be determined by location and morphology, which can have implications for their functions (Wachowiak and Shipley, 2006). While most interneurons fire traditional action potentials, many are nonspiking. These can be seen in insect olfaction (Laurent and Davidowitz, 1994; Husch et al., 2009; Tabuchi et al., 2015) and the vertebrate retina (Gleason et al., 1993). Here, we present the novel observation of nonspiking inhibitory interneurons in the antennal lobe (AL) of the adult fruit fly, *Drosophila melanogaster*. These neurons have a morphology where they innervate a patchwork of glomeruli. We used electrophysiology to determine whether their nonspiking characteristic is because of a lack of sodium current. We then used immunohistochemsitry and *in situ* hybridization to show this is likely achieved through translational regulation of the voltage-gated sodium channel gene, *para*. Using *in vivo* calcium imaging, we explored how these cells respond to odors, finding regional isolation in their responses’ spatial patterns. Further, their response patterns were dependent on both odor identity and concentration. Thus, we surmise these neurons are electrotonically compartmentalized such that activation of the neurites in one region does not propagate across the whole antennal lobe. We propose these neurons may be the source of intraglomerular inhibition in the AL and may contribute to regulation of spontaneous activity within glomeruli.

## Significance Statement

These findings are a novel discovery of nonspiking interneurons specifically in the olfactory system of adult *Drosophila melanogaster*. The role of the nonspiking characteristic of similar interneurons in other species is not fully understood. Further, the sources of specific regulatory mechanisms such as intraglomerular inhibition in the fly are unclear. The characterization of nonspiking interneurons in *Drosophila* begins to explain these mechanisms and provides an avenue for further study into the roles of similar cells across species.

## Introduction

Inhibitory local interneurons (LNs) are prevalent throughout animal nervous systems and often serve to regulate circuit function. One such example are the LNs in the antennal lobe (AL) of the fruit fly *Drosophila melanogaster*. The AL is the first olfactory relay of the fly, where olfactory receptor neurons (ORNs) with the same receptor converge (Vosshall et al., 2000; Couto et al., 2005) and synapse onto projection neurons (PNs; Stocker et al., 1990; Couto et al., 2005) in neuropil known as glomeruli. The PNs then transmit the signal to higher order brain regions (Bargmann, 2006). Odor ligands bind olfactory receptors on the antennae, coordinating spike times across the activated ORNs. PNs tend to rest at a potential close to their spiking threshold and the coordinated input from ORNs drives an odor response (Bhandawat et al., 2007; Kazama and Wilson, 2008). LNs regulate this activity through presynaptic and postsynaptic inhibition (Olsen and Wilson, 2008; Root et al., 2008; Suzuki et al., 2020).

The inhibition from LNs is important to odor coding in *Drosophila*. By inhibiting ORNs, LNs allow the full dynamic range of ORN spiking to be used across a broad range of odor concentrations (Olsen and Wilson, 2008). This leads to an increase in odor discriminability for two reasons. First, strong odors are prevented from saturating responses. And second, small differences between similar odors can be highlighted (Olsen et al., 2010). These gain and contrast controls enhance olfactory responses.

One poorly understood aspect of *Drosophila* LNs is their variety of morphologies. Previous literature has demonstrated the multitude of complex innervation patterns of LNs (Chou et al., 2010), but little is known about the function of these various populations. In mammalian olfactory bulbs (OBs), LN morphology and anatomic location in the strata of the bulb allow for distinguishing cell classes, therefore simplifying their identification and further characterization. Therefore, the roles of LN subtypes become more decipherable. In *Drosophila*, LN morphologic classifications can only be determined *post hoc*. Little information can be gained about the role of an LN by the location of its soma in proximity to the neuropil, and the AL is not organized into layers like the OB. This complicates the study of *Drosophila* LN morphology in relation to function. Physiologic characterization of LNs has revealed distinctions (Chou et al., 2010; Seki et al., 2010) and some classes of these LNs have been probed for morphology. Additionally, previous work has shown unique roles for subpopulations of LNs (Huang et al., 2010; W.W. Liu and Wilson, 2013; Suzuki et al., 2020; Chou et al., 2022; Sizemore et al., 2022). However, correlating LN physiology with morphology remains difficult. Further, there are few genetic driver lines available to specifically label morphologic classes for study.

One interesting physiological class of olfactory LNs observed in many insects outside of *Drosophila* are the nonspiking LNs (Laurent and Davidowitz, 1994; Husch et al., 2009; Tabuchi et al., 2015). These LNs do not fire action potentials like their spiking counterparts and instead rely on graded potentials for transmitter release. In the cockroach, spiking and nonspiking antennal lobe LNs have morphologic distinctions (Fusca and Kloppenburg, 2021), but the specific role of nonspiking characteristic remains unclear.

In this work, we employed the R32F10-Gal4 driver line (Jenett et al., 2012) to study the role of one such LN morphologic class in olfaction. R32F10-Gal4 labels patchy LNs (Sizemore et al., 2022) which are named so for their discontinuous, patchwork innervation pattern of glomeruli (Chou et al., 2010). The LNs labeled by this driver line have been morphologically characterized (Sizemore et al., 2022), but much remains to be learned about their physiology. We demonstrated that these LNs are akin to LN populations found in other insects: they are nonspiking. We explored this characteristic using electrophysiology, calcium imaging, and tissue staining. Our results suggest these patchy, nonspiking LNs are compartmentalized through electrotonic isolation achieved through post-transcriptional regulation of sodium channels. Lastly, we propose that these cells are potentially involved in two LN functions which are poorly understood: intraglomerular inhibition and spontaneous activity regulation.

## Materials and Methods

### Fly rearing

Male and female flies were raised and crossed in sparse cultures at 25°C on cornmeal, dextrose, and yeast medium (adapted from Brent and Oster, 1974). Crosses involving Para were performed at 20°C to prevent any effects of higher temperatures on the modified Para channels. Genetic lines used in this study can be found in Table 1. For electrophysiology experiments, adult flies 1–2 d posteclosion (dpe) were used. FlpTag and HCR experiments were performed on 5- to 7-dpe flies, and calcium imaging was done on 7- to 9-dpe flies.

**Table 1:** Genetic lines of flies used

Fly Line	Source	Identifier
GMR32F10-Gal4	Bloomington	RRID:BDSC_49725
GMR70A09-Gal4	Bloomington	RRID:BDSC_47720
UAS-mCD8::GFP	Bloomington	RRID:BDSC_32194
GMR32F10-LexA	Bloomington	RRID:BDSC_53565
LexAop-GFP	Bloomington	RRID:BDSC_52266
UAS-FLP	Bloomington	RRID:BDSC_4539
para-flptag-GFP	Bloomington	RRID:BDSC_92146
NP3056-Gal4	DGRC	RRID:113080
UAS-jGCAMP7b	Bloomington	RRID:BDSC_79029
UAS-mCherry	Bloomington	RRID:BDSC_52268
LexAop-mCherry	Bloomington	RRID:BDSC_52271

Transgenic *Drosophila* lines used in this study from the Bloomington Drosophila Stock Center and the Drosophila Genomics Resource Center.

### Odors and delivery

Odorants, dilutions, and solvents used in experiments for each figure are listed in Table 2. During electrophysiology and calcium imaging recordings, odors were delivered via an olfactometer. A 2.2 l/min carbon-filtered airstream was constantly presented to the fly. To deliver odors, 0.2 l/min of this stream was redirected through an odor vial, further diluting the odor by 10-fold. Each odor presentation throughout the study lasted 0.5 s.

**Table 2:** Odorants and dilutions in each figure

Figure	Odorant	Dilution	Solvent	Source	Identifier
1	Pentyl acetate	10^−2^, 10^−4^	Parraffin oil	Sigma-Aldrich	CAS: 628-63-7
4, 5	Pentyl acetate	10^−4^	Parraffin oil	Sigma-Aldrich	CAS: 628-63-7
4, 5	Benzaldehyde	10^−4^	Parraffin oil	Sigma-Aldrich	CAS: 100-52-7
4, 5	cVA	Pure	None	Pherobank	CAS: 6186-98-7
4, 5	Valeric acid	10^−4^	Water	Sigma-Aldrich	CAS: 109-52-4
6	Pentyl acetate	10^−10^, 10^−8^, 10^−6^, 10^−4^	Parraffin oil	Sigma-Aldrich	CAS: 628-63-7

Chemicals, dilutions, solvents, and sources of odors used during odor exposure experiments.

### Electrophysiology

Whole-cell patch-clamp recordings were done *in vivo*. Flies were mounted in a custom foil chamber and the brain was exposed. External saline solution was constantly perfused throughout each experiment and contained (in mm): 103 NaCl, 3 KCl, 5 N-tris(hydroxymethyl)methyl-2-aminoethane-sulfonic acid, 8 trehalose, 10 glucose, 26 NaHCO_3_, 1 NaH_2_PO_4_, 1.5 CaCl_2_, and 4 MgCl_2_ (adjusted to 270–275 mOsm). This solution was bubbled with 95% O_2_/5% CO_2_ and adjusted to a pH of 7.3. Pipettes were pulled to 7–11 MΩ resistance from thin-walled borosilicate glass (World Precision Instruments; 1.5-mm outer diameter, 1.12-mm inner diameter). For current clamp recordings, pipettes were filled with an internal solution containing (in mm) 140 potassium aspartate, 10 HEPES, 4 MgATP, 0.5 Na_3_GTP, 1 EGTA, and 1 KCl. For voltage clamp recordings, the internal solution contained (in mm) 140 CsOH, 140 aspartic acid, 10 HEPES, 1 EGTA, 1 CsCl, four MgATP, and 0.5 Na_3_GTP. Internal solution osmolarities were adjusted to 265 mOsm with a pH of 7.2. Preparations were illuminated during patching via a fiber-optic oblique infrared LED (Thorlabs), and cells were targeted by their expression of mCD8::GFP under binary expression systems. All recordings were digitized at 10 kHz. Current clamp recording data were low pass filtered at 5 kHz with an AM Systems model 2400 amplifier (AM Systems). After breaking in, resting potentials of spiking cells were adjusted to elicit baseline firing rates observed in previous cell-attached recordings. Nonspiking cells were adjusted to similar potentials. Voltage clamp data were low pass filtered at 1 kHz and cells were held at −60 mV. Current signals were conditioned using positive/negative subtraction (Molleman, 2002). Positive/negative subtraction, also known as leak subtraction, utilizes a series of small depolarizing voltage steps which evoke capacitive transients and background/leak conductances but fail to produce active current responses. These passive components scale with the size of the voltage step. A larger voltage step equal to the sum of the smaller steps is then applied to evoke any active conductances. The passive components from the small voltage steps are summated and then subtracted from the current response to the larger voltage step. The resulting current response then only contains active conductances such as those from voltage-gated channels. This signal correction was applied offline, such that raw signals were preserved. Voltage-gated sodium currents were distinguished by their sensitivity to tetrodotoxin (TTX).

### Pharmacology

To determine sodium-dependent spikes and currents, TTX (1 μm; Alomone Labs) was used to specifically block voltage-gated sodium channels. During voltage clamp recordings, synaptic transmission was blocked with CGP-54 626 (50 μm, Tocris, CAS: 149184-21-4), mecamylamine (100 μm, Sigma, CAS: 826-39-1), and picrotoxin (5 μm, Sigma, CAS: 124-87-8). Experiments involving these drugs were done using a recirculating perfusion system.

### Immunohistochemistry (IHC)

**Figure 1.**
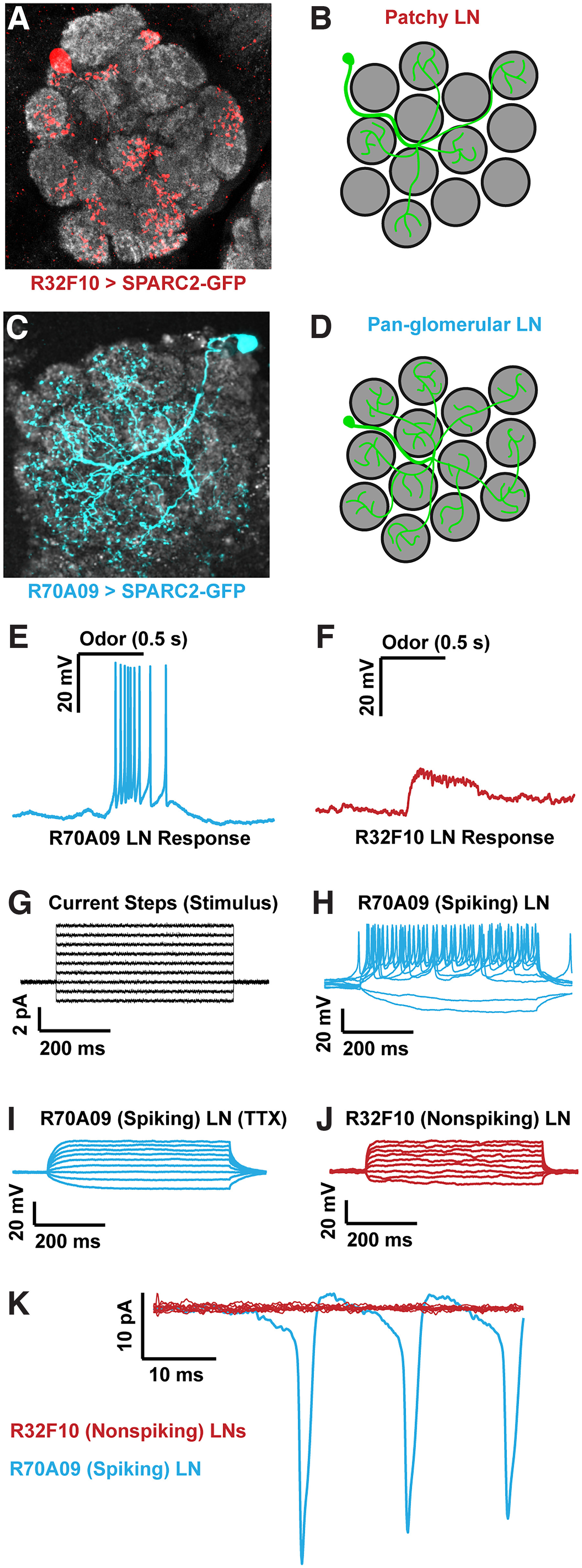
Population of nonspiking LNs has patchy morphology and lacks voltage-gated sodium current. Innervation patterns of a single LNs labeled by the (***A***) R32F10-Gal4 and (***C***) R70A09-Gal4 driver lines obtained by stochastic labeling using SPARC2 stochastic labeling (red and blue, respectively). Single cells are stochastically labeled from the Gal4 populations. ***B***, ***D***, Cartoon examples of the patchy and pan-glomerular LN morphologies, respectively. Sample LN responses to 10^−4^ pentyl acetate for (***E***) R70A09-Gal4 and (***F***) R32F10-Gal4 lines. Horizontal bar denotes the timing of the odor pulse as well as scale (500 ms). Other odors tested include methyl acetate and ethyl acetate at 10^−4^ and 10^−2^ dilutions, respectively. ***G***, Current step stimulus applied to sample traces in ***H–J***. ***H***, Spiking LN voltage response to current clamp steps from ***G***. ***I***, Same as in ***H***, except in the presence of 1 μm TTX. ***J***, Voltage response to steps in ***G*** for a sample nonspiking LN. ***K***, Voltage clamp responses of a single spiking LN (blue) and multiple nonspiking LNs (red, *n* = 9) during a voltage step from −60 mV holding potential to −30 mV for a duration of 50 ms. Horizontal bars denote time, vertical bars denote voltage or current.

**Figure 2.**
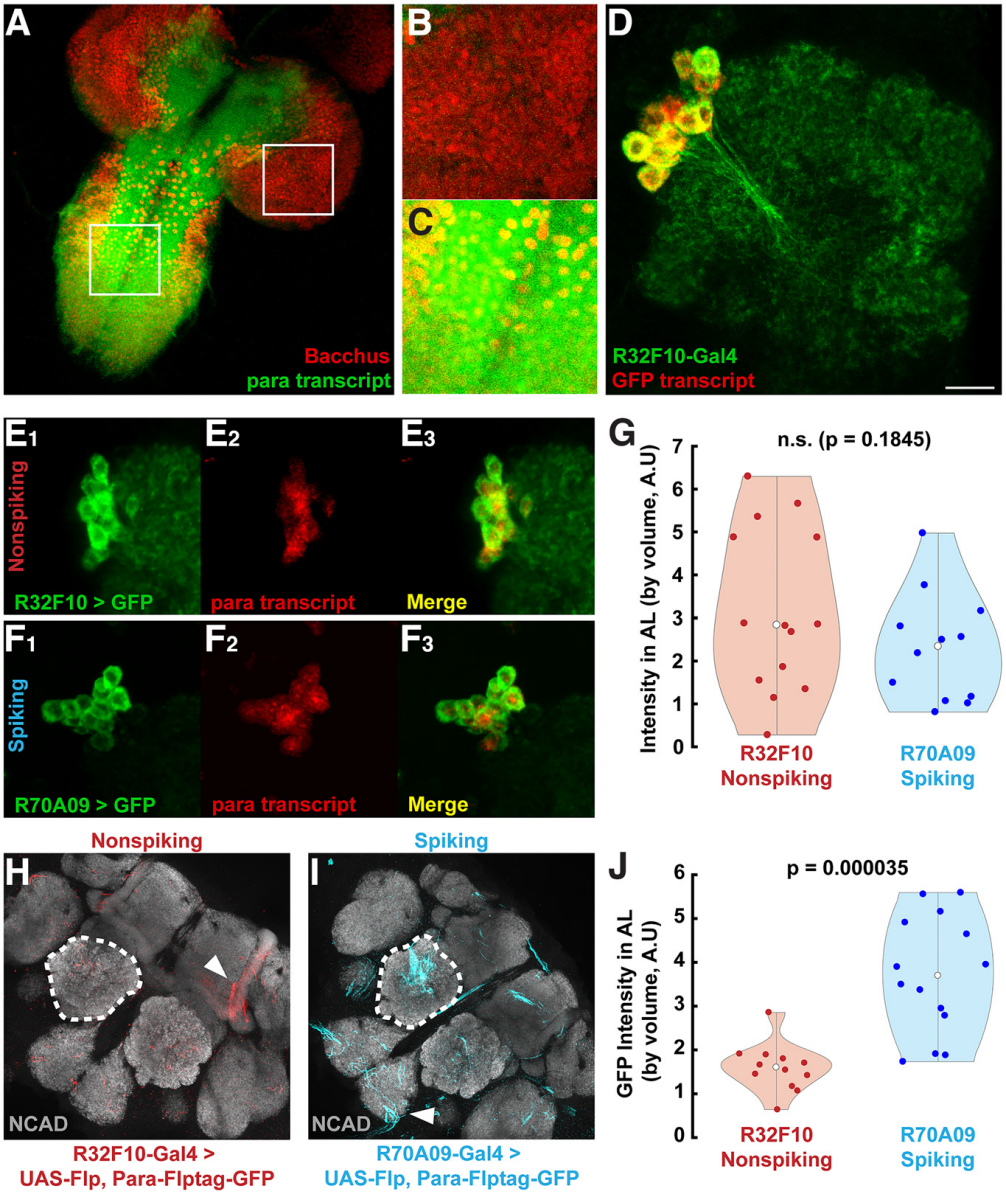
Voltage-gated sodium channel gene transcript, *para*, is detected in adult nonspiking LNs but conditional tagging reveals lack of translation. ***A***, Larval central nervous system (CNS) expression of *para* transcript revealed through HCR. White boxes indicate regions of interest expanded in B and C and also represent scale at 63 μm along each side. Red indicates Bacchus protein which labels most CNS neuron nuclei. Transcripts of *para* are indicated in green. ***B***, ***C***, Expanded views of brain and ventral nerve cord, respectively, as indicated in ***A***. Transcript was rarely detected in brain regions labeled with Bacchus while overlap was common in the ventral nerve cord (*n* = 4). ***D***, HCR straining for GFP transcript (red) in R32F10-GFP>UAS-mCD8::GFP (GFP in green). GFP transcript stain was only detected in GFP-positive cells. Scale bar indicates 10 μm. ***E***, ***F***, Sample GFP (left), *para* transcript (middle), and merge (right) of (***E***) nonspiking and (***F***) spiking LN populations in the AL. Transcripts were stained using hybridization chain reaction (Molecular Instruments), and images were masked to emphasize co-labeling with GFP. ***G***, Violin plot of *para* transcript stain intensities in the AL, normalized by volume. *n* = 14 for R32F10-Gal4, 12 for R70A09. White circles denote means. Difference is not statistically significant (Student’s *t* test, *p* = 0.18). Sample images for (***H***) R32F10-Gal4 and (***I***) R70A09-Gal4 of UAS-Flp-driven para-FlpTag-GFP (red and blue, respectively). Dashed lines indicate measured AL regions; arrowheads indicate examples of non-AL staining. Background stain (anti-NCAD) is shown in gray. ***J***, Violin plot of GFP labeling intensity, normalized by AL volume. *n* = 12 for R32F10-Gal4, 14 for R70A09. White circles denote means. Difference is significant (Student’s *t* test, *p* = 0.000035).

Brains were dissected in ice cold external saline and immediately fixed for 15 min in 4% paraformaldehyde, then blocked in PBS containing 2% Triton X-100 and 10% normal goat serum for 30 min, both at room temperature. Brains were then incubated in primary antibody solutions for 1–2 d at 4°C and secondary antibody solutions for 2–12 h at room temperature. Primary and secondary antibodies and dilutions are listed in Table 3. Samples were continuously protected from light starting after dissection. A Zeiss LSM710 confocal microscope was used to acquire images under 40× or 63× magnification with 0.5-μm optical section depth. Images shown are z-projections generated in ImageJ. Quantification was performed in ImageJ and statistical analysis was performed in MATLAB (Table 4). Immunohistochemistry (IHC) was performed to amplify the relatively weak and stochastic labeling techniques in Figures 1 and 2. To account for stochastic labeling in the FlpTag experiments, we only measured signals from the right AL of each fly regardless of stain intensity. FlpTag recombination was verified in each fly by GFP expression outside the AL. Flies without FlpTag-GFP expression outside the AL were excluded.

**Table 3:** Antibodies and other tissue staining reagents

Reagent	Dilution	Source	Identifier
Mouse anti-Bruchpilot	1:50	Developmental Studies Hybridoma Bank, Iowa	Catalog #nc82; RRID: AB_2314866
Rat anti-NCAD	1:500	Developmental Studies Hybridoma Bank, Iowa	Catalog #DN-Ex #8; RRID: AB_528121
Chicken anti-GFP	1:1000	Invitrogen	Catalog #A-10262; RRID: AB_2534023
Rabbit anti-DsRed	1:400	Clontech	Catalog #632496; RRID: AB_10013483
Streptavidin Alexa Fluor 568	1:250	Invitrogen	Catalog #S11226; RRID: AB_2315774
Goat anti-chicken Alexa Fluor 488	1:250	Invitrogen	Catalog #A-11039; RRID: AB_2534096
Goat anti-rabbit Alexa Fluor 633	1:250	Invitrogen	Catalog #A-21071; RRID: AB_2535732
Goat anti-rat Alexa Fluor 568	1:250	Invitrogen	Catalog #A-11077; RRID: AB_2534121
Goat anti-mouse Alexa Fluor 633	1:250	Invitrogen	Catalog #A-21050; RRID: AB_2535718
HCR fruitfly-para-B1	1:250	Molecular Instruments	N/A
HCR transgenic-EGFP-B3	1:250	Molecular Instruments	N/A
HCR amplifier B1 Alexa Fluor 647	1:50	Molecular Instruments	N/A
HCR amplifier B3 Alexa Fluor 546	1:50	Molecular Instruments	N/A
Biocytin Hydrazide	13 mm	Life Technologies	Catalog #B-1603

Primary and secondary antibodies and *in situ* hybridization reagents and sources.

**Table 4:** Statistical tests and parameters

Figure	Distribution	Test	Sample size	Statistical data
2*G*	Normal	Unpaired *t* test (two tailed)	R32F10 *n* = 14, R70A09 *n* = 12	*t* = −1.3664, df = 24, *p* = 0.1845
2*J*	Normal	Unpaired *t* test (two tailed)	R32F10 *n* = 12, R70A09 *n* = 14	*t* = 5.0714, df = 24, *p* = 0.000035
4*G*	Normal	ANOVA (Tukey’s *post hoc* test)	NP3056 *n* = 8, R32F10 *n* = 15, R70A09 *n* = 11	Latency *p* = 0.3484 half rise *p* = 0.1807 half decay *p* = 8.8e-4 response duration *p* = 6.2e-4
5*D*	Normal	ANOVA	NP3056 *n* = 8, R32F10 *n* = 15, R70A09 *n* = 10	*F* = [35.5101, 18.5213, 28.5431, 21.8342]; *p* = [1.6e-8, 5.8e-6, 1.1e-7, 1.4e-6]
5*G*	Nonparametric	Kruskal–Wallis	NP3056 *n* = 8, R32F10 *n* = 15, R70A09 *n* = 11	Descending, left to right: X2 = [16.3083, 11.1783, 1.3186, 19.2841, 11.6920, 9.0253]; *p* = [2.9e-4, 0.0037, 0.5172, 6.5e-5, 0.0029, 0.011]
6*D*	Normal	ANOVA (Tukey’s *post hoc* test)	NP3056 *n* = 11, R32F10 *n* = 10, R70A09 *n* = 12	R70A09: *F* = 0.5698, *p* = 0.6383; NP3056: *F* = 1.1598, *p* = 0.3374; R32F10: *F* = 7.4735, *p* = 0.00005; R32F10 concentration pair *p* values: −10 and −8 = 0.9677; −10 and −6 = 0.0379; −10 and −4 = 0.0013; −8 and −6 = 0.1047; −8 and −4 = 0.0049; −6 and −4 = 0.5954

Summary of statistics used in each figure

### *In situ* hybridization

Hybridization chain reaction (HCR; Molecular Instruments) was used to detect RNA transcripts. Probes for *para* and GFP transcripts were engineered by Molecular Instruments. Amplification hairpins used can be found in Table 3. The “HCR RNA-FISH protocol for sample in solution” was adapted for our samples by making the following changes: 500-μl volumes were adjusted to 200 μl (concentrations were kept the same), and Triton X-100 was substituted for Tween 20. Samples were continuously protected from light after dissection. Imaging was performed the same as immunohistochemistry. Endogenous fluorescence from GFP expressed in the cells of interest was also imaged in these samples. Images shown in Figure 2*A*,*B* are masked to enhance visualization of the *para* transcript signal and were generated in ImageJ. Quantification was performed in ImageJ and statistical analysis was performed in MATLAB (Table 4).

### Wide-field calcium imaging

GCAMP7b (Dana et al., 2019) was expressed under Gal4/UAS control and odor responses were acquired using wide-field imaging. Recordings were done on an Olympus BX51WI microscope under an Olympus 40× magnification objective (1-U2M587, Olympus). Samples were illuminated using a 470-nm blue LED (M470L4, Thorlabs) and images were captured at 20 frames per second using a Photometrics Prime CMOS camera (Photometrics). A shutter (SH1, Thorlabs) was used to allow the LED intensity to equilibrate and prevent light exposure between trials. Two-minute intertrial intervals were used to allow baseline fluorescence level stabilization and to prevent phototoxicity. Video recordings were motion corrected using the “Stackreg” plugin in ImageJ. Changes in fluorescence (ΔF/F) were calculated as the difference between a given frame and the average baseline, normalized to the baseline and analyses were performed in MATLAB (Table 4). Values were measured across the entirety of the AL visible in the selected focal plane unless indicated otherwise, and pixels outside the AL were masked for analysis. Peak odor responses were defined as the average of three frames centered on the highest ΔF/F value in the odor period. Resulting peak ΔF/F frames were then averaged across three trial repeats and Gaussian low-pass filtered at 10 × 10 pixels.

For temporal analysis of calcium imaging data, we calculated four metrics; response latency, half rise time, half decay time, and response duration. The response latency was defined as the time between odor onset and the first peak in the derivative of the ΔF/F trace during the odor period. Half rise time was the time between the response onset as determined in the latency calculation and when the ΔF/F value was half of the peak ΔF/F value. Half decay time was the duration in which the ΔF/F value remained above half of the peak value after reaching the peak. Response duration was calculated as the time between the ΔF/F values determined as the half rise and half decay.

Because the focal plane was not always consistent between preparations, principal components analysis (PCA) and correlation coefficients were calculated within individual fly responses and then compared across flies. For input into principal components analysis and correlation calculations, the average peak ΔF/F frames were concatenated row-by-row from the frame’s pixels into 1-dimensional vectors for each fly. The resulting vectors each had the same number of elements as pixels in the average frames. These vectors were then inserted into a matrix for analysis for each individual fly, with columns representing trials and rows representing corresponding pixels. The explained variance in PCA and correlation coefficients were then compared between flies. The images are sample PC scores reshaped into the dimensions of the input images. For correlation coefficients, ΔF/F values were normalized as *z* scores to reduce the contribution of response strength to the correlation, thus biasing the result to focus on activation patterns.

## Results

### A population of local interneurons in the antennal lobe does not fire sodium-dependent action potentials

We began characterizing patchy LNs by measuring their odor responses using whole-cell patch-clamp electrophysiology. To target specific cells, we used the R32F10-Gal4 driver line (Jenett et al., 2012), labeling only patchy LNs within the AL (Fig. 1*A*,*B*; Sizemore et al., 2022). Another driver line, R70A09-Gal4 (Jenett et al., 2012), labels mainly pan- and nearly pan-glomerular LNs (Fig. 1*C*,*D*; Suzuki et al., 2020). These LNs display robust responses to most odors characterized by a burst of large spikes paired with odor onset (Fig. 1*E*). Surprisingly, we failed to observe action potentials when recording from the R32F10-Gal4 labeled LNs, despite a clear odor-evoked depolarization (Fig. 1*F*). This phenomenon persisted across holding potentials and broadly activating odors, in addition to a lack of spikes during spontaneous activity. To determine whether this lack of spiking was simply an artifact of recording or choice of odor, we stimulated cells with an array of current injections (Fig. 1*G*). In R70A09-Gal4 cells, each positive current injection reliably elicited strong spiking responses with larger steps eliciting stronger responses (Fig. 1*H*), which were abrogated in the presence of the voltage-gated sodium channel blocker, tetrodotoxin (TTX; Fig. 1*I*). However, the same current injection stimuli did not evoke spikes in R32F10-Gal4 patchy LNs (Fig. 1*J*). Qualitatively, the voltage response to current injection of these nonspiking LNs resembled that of spiking LNs in TTX. Next, we examined whether patchy R32F10-Gal4 LNs exhibit sodium current, but simply not enough to elicit action potentials. To investigate this possibility, we performed voltage clamp recordings. We determined the TTX-sensitive sodium currents by recording during voltage steps before and after TTX application. These currents can be attributed solely to voltage-gated sodium channels. While *Drosophila* LNs are not well suited to achieve strong space clamp, we were still able to observe TTX-sensitive sodium currents reliably in spiking R70A09-Gal4 LNs (Fig. 1*K*). Performing the same experiment on R32F10-Gal4 LNs, we failed to observe any TTX-sensitive currents in the cells (Fig. 1*K*). Therefore, we did not detect action potentials nor sodium current in R32F10-Gal4 patchy LNs in the AL and thus demonstrate the existence of nonspiking LNs in the fly antennal lobe.

### Nonspiking LNs do not have voltage-gated sodium channels but still transcribe the *para* gene

*Drosophila* have one voltage-gated sodium channel gene, *para*, and these channels are required for firing traditional action potentials (Germeraad et al., 1992). Mutations in *para* tend to be lethal or debilitating to the fly, demonstrating the importance of sodium current for proper neuronal function (Siddiqi and Benzer, 1976). However, it has been observed through single-cell sequencing that most central brain neurons in *Drosophila* larvae lack *para* expression, while adult brains express *para* in nearly all neurons (Ravenscroft et al., 2020). Therefore, we first probed for the presence of *para* gene products to confirm the absence of sodium current in LNs which we observed electrophysiologically. Because of widespread *para* expression, we performed *in situ* hybridization to spatially probe for *para* transcript. Because it is possible *para* was expressed in our cells of interest but only in small quantities, we used hybridization chain reaction (HCR, Molecular Instruments) to amplify and detect potentially weak transcript signals. We verified our probe’s specificity for *para* transcripts by staining *Drosophila* larval central nervous systems (Fig. 2*A*), in which the brain largely does not transcribe *para* (Fig. 2*B*), as opposed to the ventral nerve cord which does transcribe the gene (Ravenscroft, 2020; Fig. 2*C*). GFP transcripts can be detected in the AL of adult R32F10-Gal4 flies (Fig. 2*D*). With HCR, *para* transcript was detected in the somas of both spiking (Fig. 2*F*) and nonspiking (Fig. 2*E*) LNs. The amount of signal was comparable between R32F10-Gal4 and our control line R70A09-Gal4 (Fig. 2*G*). This driver line was chosen as a control here and for following experiments because it displays prototypical spiking responses and does not label any cells with the patchy morphology (Suzuki et al., 2020).

Transcript detection does not equate directly to gene expression (Becker et al., 2018). Post-transcriptional regulation serves a role in controlling the amount of protein product of a gene. Given the results in Figure 2*E–G*, there are two possible scenarios: first, *para* undergoes post-transcriptional regulation in these nonspiking LNs; or second, Para channels are produced but are undetectable using electrophysiology. To test these possibilities, we used the FlpTag approach (Fendl et al., 2020). FlpTag utilizes a conditional labeling of Para with GFP driven by the expression of UAS-Flp under the control of Gal4 lines, therefore restricting staining to only the cells of interest. Staining in regions either inside or outside of the ALs indicated successful FlpTag reactions and only brains in which this was observed were included. Para densely localizes to the presumed action potential initiation site of *Drosophila* neurons (Ravenscroft et al., 2020). We observed the characteristic clustering of Para signal observed in previous literature (Fig. 2*H*,*I*; Ravenscroft et al., 2020). Although our implementation showed stochasticity in labeling between ALs of the same brain, we detected heavy Para-FlpTag staining in spiking LNs labeled by R70A09-Gal4 (Fig. 2*I*). We observed less Para-FlpTag staining in nonspiking LNs labeled by R32F10-Gal4 (Fig. 2*H*) and these differences in Para signal were significant (Fig. 2*J*). Taken together, these results indicate nonspiking LNs indeed do not express Para channels, despite continued transcription of the gene.

### Calcium imaging of nonspiking LNs reveals variable spatial patterns of activation across odors

**Figure 3.**
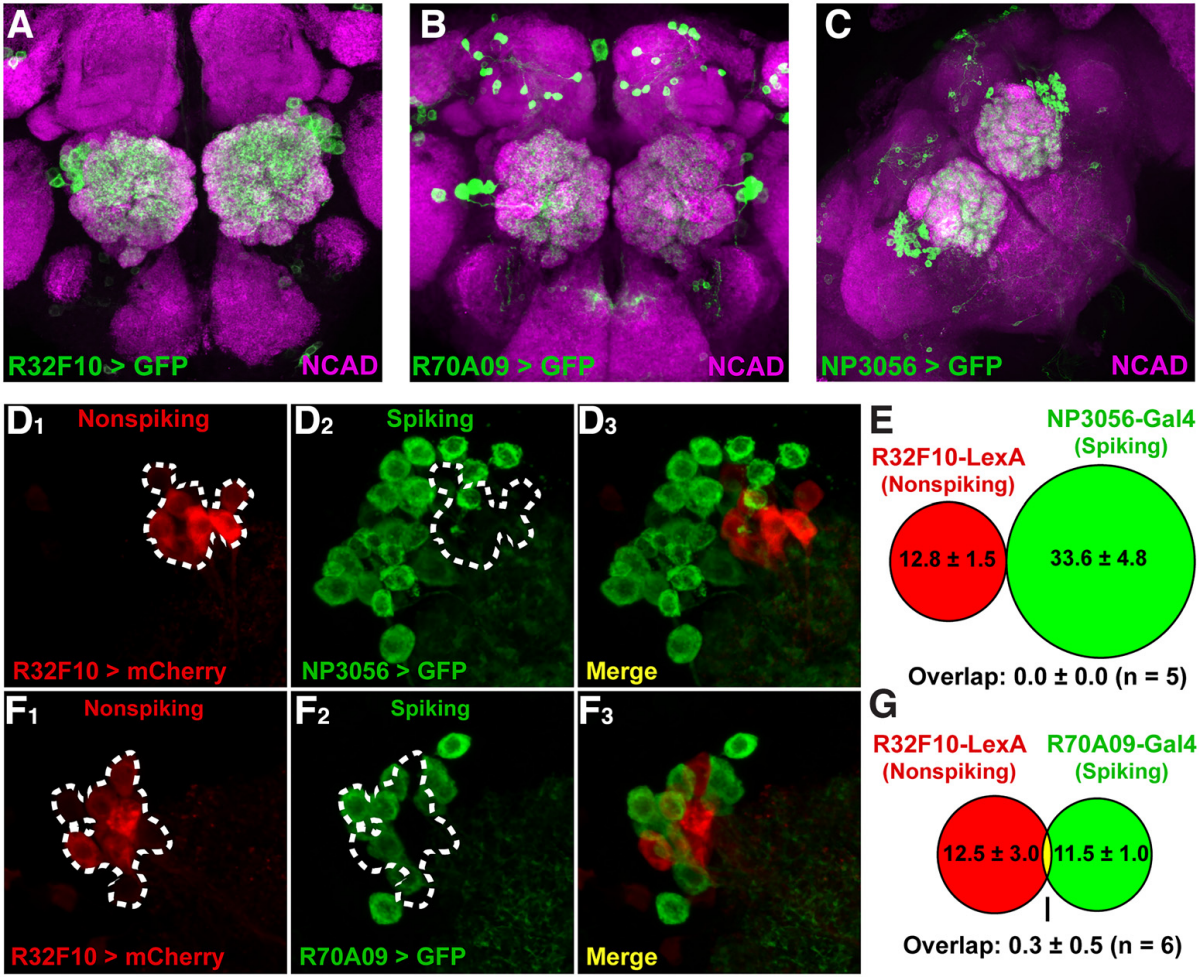
Nonspiking LNs labeled by R32F10 are a distinct population of neurons. Full brain expression patterns of (***A***) R32F10-Gal4, (***B***) R70A09-Gal4, and (***C***) NP3056-Gal4 expressing GFP (green). Background stain is anti-NCAD (magenta). Sample images of R32F10-LexA > LexAop-mCherry (***D1***, ***F1***), NP3056-Gal4 > UAS-GFP (***D2***), R70A09-Gal4 > UAS-GFP (***F2***), and merges (***E3***, ***G3***). White dashed lines denote the location of R32F10-LexA labeled somas. Quantification indicates effectively zero overlap between R32F10-Lexa and (***E***) NP3056-Gal4 (*n* = 5) and (***G***) R70A09-Gal4 (*n* = 6).

To assess the physiology of nonspiking patchy LNs in *Drosophila*, we performed wide-field calcium imaging of LN populations during odor responses. We compared responses between the R32F10-Gal4 nonspiking LNs (Fig. 3*A*), R70A09-Gal4 spiking LNs (Fig. 3*B*), and the broader LN-labeling NP3056-Gal4 line (Fig. 3*C*), each expressing UAS-GCAMP7b. R70A09-Gal4 labels a similar number of LNs as R32F10-Gal4 (Fig. 3*G*), and NP3056-Gal4 is a widely used and well characterized driver line which represents most identified morphologic classes. As shown in previous literature, NP3056-Gal4 and R70A09-Gal4 have little to no overlap in cells labeled (Suzuki et al., 2020), and NP3056 LNs generally exhibit action potentials (Chou et al., 2010). R32F10-LexA, which shares a promoter fragment with R32F10-Gal4 and labels many of the same cells (Sizemore et al., 2022), also has little to no overlap with NP3056-Gal4 (Fig. 3*D*,*E*) and R70A09-Gal4 (Fig. 3*F*,*G*), indicating these LN populations are mostly distinct.

**Figure 4.**
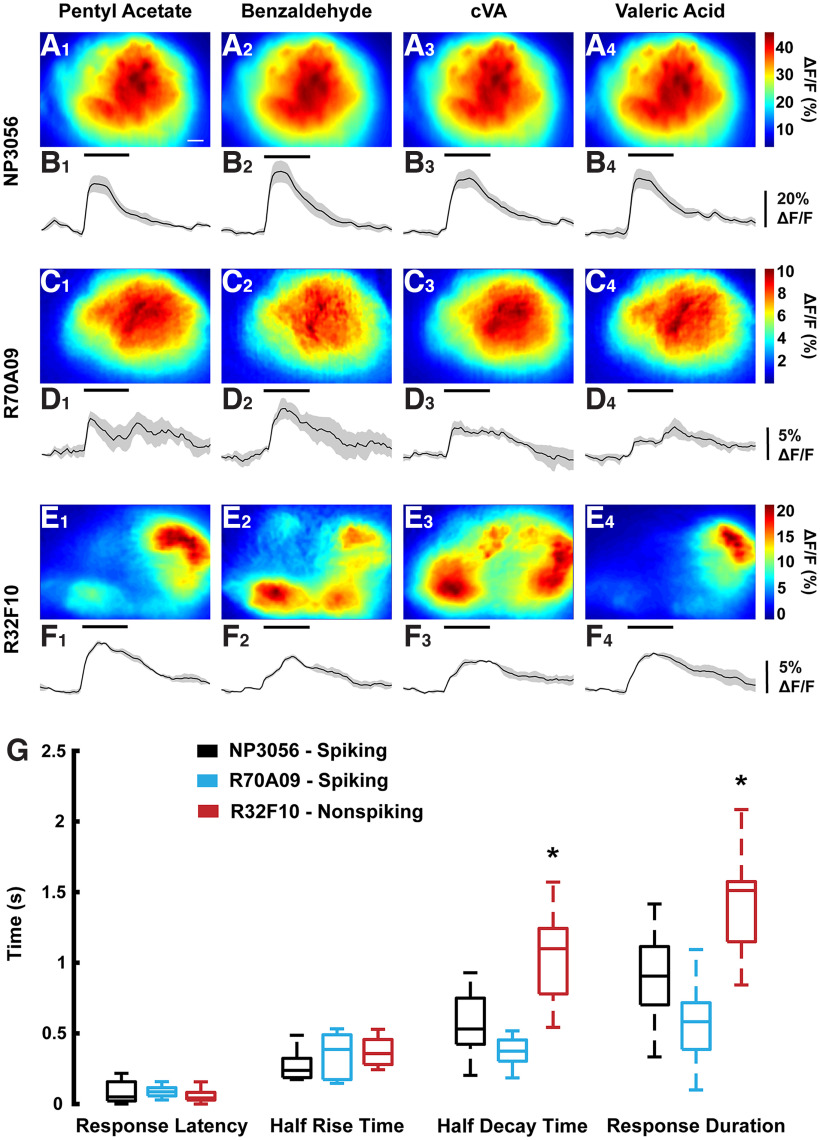
Sample odor activation patterns of LN lines reveal specific responses in nonspiking cells. NP3056-Gal4 (***A1–A4*** and ***B1–B4***), R70A09-Gal4 (***C1–C4*** and ***D1–D4***), and R32F10-Gal4 (***E1–E4*** and ***F1–F4***) > UAS-GCAMP7b response patterns and temporal ΔF/F traces to pentyl acetate, benzaldehyde, cVA, and valeric acid. Images are an average of the three frames around the peak of the ΔF/F in response to a given odor and are normalized to the same scale within a given fly. Colorbars indicate the range of ΔF/F values in percent. Traces represent the average of three trials ± SEM. Vertical bars denote ΔF/F value in percent. Horizontal bar denotes the timing of the odor pulse as well as scale (500 ms). Odors were presented at 10^−4^ dilution in the odor vial except for cVA, which was pure. Scale bar in ***A1*** represents 10 μm. Images and traces each correspond to the same fly, e.g., one fly was used to generate ***A1–A4*** and ***B1–B4***. ***G***, Response latency, half rise time, half decay time, and response duration of odor responses average across flies and odors. Central lines indicate means while the top and bottom edges of the boxes indicate 75th and 25th percentiles, respectively. The whiskers represent the range from minimum to maximum, excluding outliers. Outliers were excluded from plotting but included in analysis. R32F10-Gal4 was significantly different from the other lines for half decay time and response duration (denoted by *, ANOVA with Tukey’s *post hoc* test, *p* = 8.8e-4 and 6.2e-4, respectively).

We first presented a panel of four odors at a 10^−4^ dilution, chosen to represent both odors which broadly activate many ORs (pentyl acetate, benzaldehyde) and odors which activate a restricted subset of ORs (cVA, valeric acid), with varying degrees of similarity in activated glomeruli. Peak responses were selected as the highest ΔF/F value during the odor presentation period and averaged from the three frames centered around the peak. We hypothesized that within each fly, spiking lines would respond with the same spatial pattern for each odor, as action potentials would propagate their activity across the entire antennal lobe. For nonspiking LNs, we hypothesized that activation may be spatially restricted as the graded potentials would not propagate as actively. This would result in activation patterns which would spatially vary across odors within a given fly. Consistent with previous literature, the NP3056-Gal4 and R70A09-Gal4 spiking lines produced calcium responses that largely activated the entire visible region of the AL (Fig. 4*A*,*C*; Hong and Wilson, 2015), while the patchy R32F10-Gal4 cells displayed spatially variable patterns of activation across odors within each fly (Fig. 4*E*). The temporal responses of all three lines were also compared (Fig. 4*B*,*D*,*F*). We did not observe significant differences in response latency or half rise time, but R32F10-Gal4 responses had significantly longer half decay times and response durations (Fig. 4*G*). While wide-field imaging can suffer from signal contamination originating from outside the focal plane, we do not believe such signals alter these results demonstrating that nonspiking local interneurons show spatially variable responses to unique odors. Our results using wide-field imaging in spiking LN populations are also corroborated by previous literature using two-photon imaging (Hong and Wilson, 2015).

**Figure 5.**
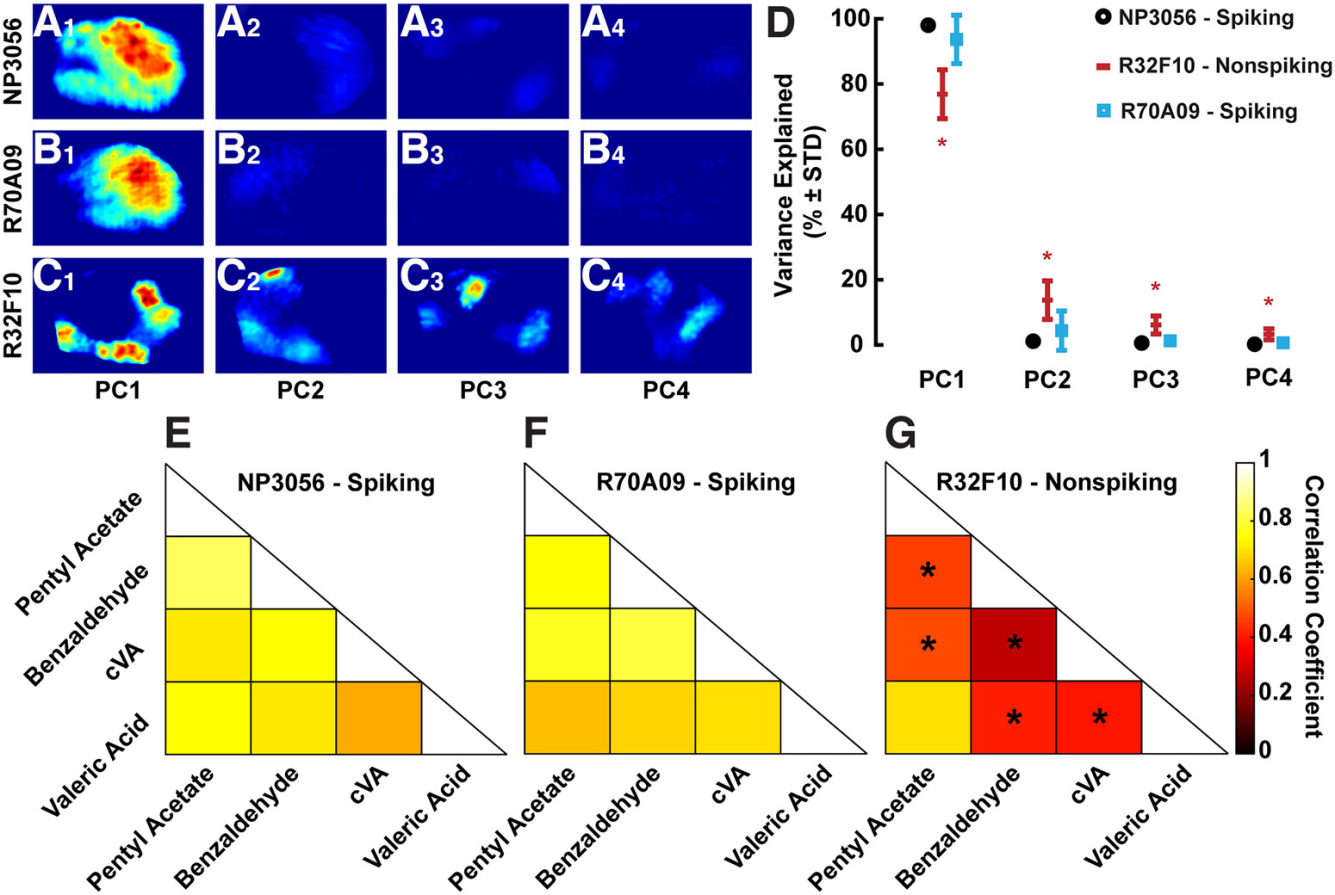
Nonspiking LN odor response patterns requires more PCs to explain comparable variance. Odor response patterns in nonspiking LNs are decorrelated. PCA was run across odor response patterns for individual flies and results for a single fly are shown here. Score outputs are projected back onto the AL to visualize sample PCs in the context of odor response spatial patterns. PC1 shows substantial contribution to the variance explanation in NP3056-Gal4 (***A1***) and R70A09-Gal4 (***B1***), while PCs 2–4 show little patterning (***A2–A4*** and ***B2–B4***). All PCs display scores in distinct spatial patterns for R32F10-Gal4 (***C1–C4***). ***D***, Mean variance explained by each PC (% ± SD), *n* = 8 for NP3056-Gal4, 15 for R32F10-Gal4, and 10 for R70A09-Gal4 lines. PCA was performed on each fly individually, and explained variances were pooled for plotting and statistical analysis. In each PC, the explained variance for R32F10-Gal4 is significantly different from the other lines (denoted by *, ANOVA with Tukey’s *post hoc* test, *p* = 1.6e^−8^, 5.8e^−6^,1.1e^−7^,1.4e^−6^ for PCs 1–4, respectively). Correlation coefficients were calculated between odor responses for (***E***) NP3056-Gal4, (***F***) R70A09-Gal4, and (***G***) R32F10-Gal4 lines. Coefficients were calculated between odors for individual flies and are presented as averages. * denotes statistical significance in the difference between a given odor pair and the corresponding odor pairs of the other two LN lines (Kruskal–Wallis with Dunn’s *post hoc* test, *p* = 2.9e^−4^ for pentyl acetate and benzaldehyde, *p* = 3.7e^−3^ for pentyl acetate and cVA, *p* = 0.52 for pentyl acetate and valeric acid, *p* = 6.5e^−5^ for benzaldehyde and cVA, *p* = 2.9e^−3^ for benzaldehyde and valeric acid, and *p* = 0.011 for cVA and valeric acid).

To quantify the spatial differences, we first ran principal components analysis (PCA) on the odor response ΔF/F activation patterns. As the focal plane in wide-field imaging is not consistent between preparations, we ran PCA on individual fly response patterns on a pixel-by-pixel basis. The percentage of variance explained by each principal component (PC) could then be averaged across flies. For individual flies, we saw only one major pattern in PC1 when PC scores were projected back onto the AL for the spiking lines (Fig. 5*A*,*B*) while we observed distinct differences in the patterns in PCs 1–4 for R32F10-Gal4 (Fig. 5*C*). These patterns represent activated regions most shared between odors. Looking at the percent of the variance explained by the PCs for each line, we saw that nonspiking cells required more PCs to explain comparable amounts of variance when compared with spiking lines (Fig. 5*D*). This indicated there was more variability in the pattern of activation across odors for nonspiking LNs than in spiking LNs.

Next, we compared how similar odor response patterns were within each fly by calculating the spatial pattern correlation coefficients between odors for each fly. Intensities were normalized to calculate only the difference in pattern regardless of the response strength. For spiking LN lines, we saw the response patterns had high correlation between odors, meaning the responses were all spatially similar (Fig. 5*E*,*F*). Coefficients for nonspiking patchy LNs were significantly lower when compared with the same odor pairings in the spiking lines (Fig. 5*G*), except for the pentyl acetate/valeric acid pair. These results suggest nonspiking LNs do not activate across the entire antennal lobe as is observed in spiking LN lines, and that the pattern of activation is specific to the presented odor.

### Nonspiking LN response patterns vary across concentrations of an odor

**Figure 6.**
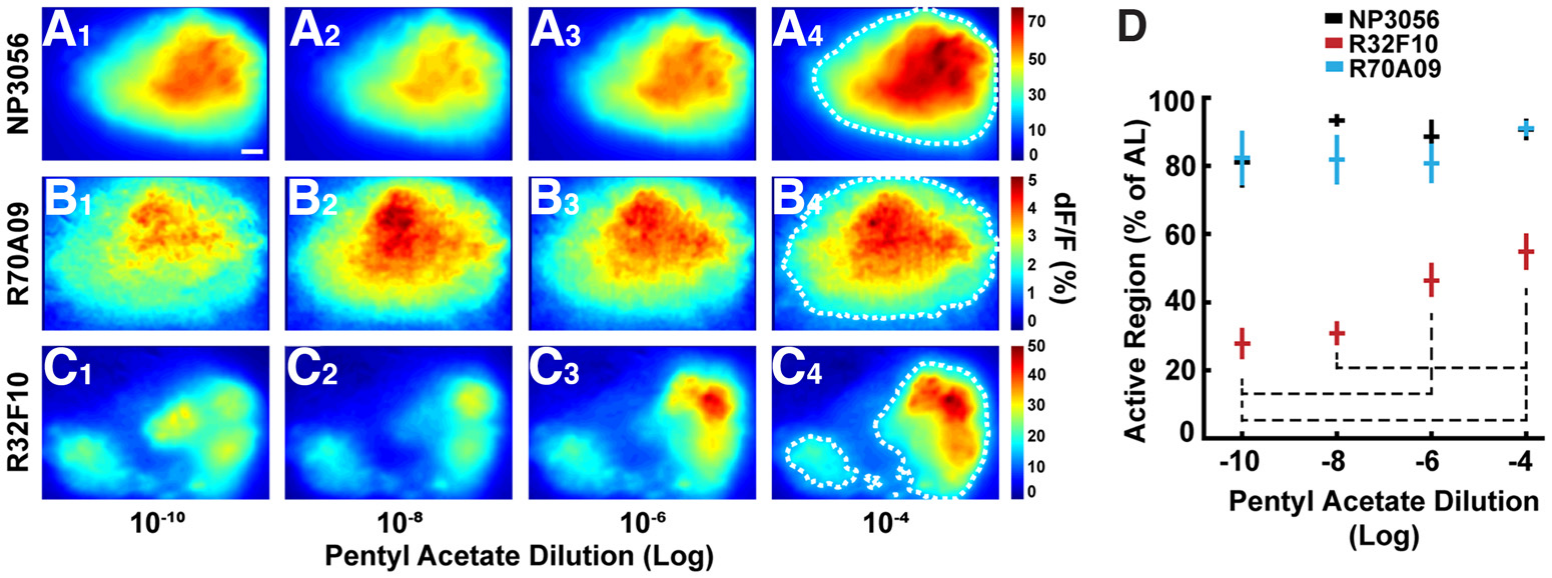
Sizes of activated regions increase with odor concentration in nonspiking LNs. ***A–C***, Same as Figure 4, but for increasing concentrations of pentyl acetate. Odor concentrations were 10^−10^, 10^−8^, 10^−6^, and 10^−4^. Scale bar in ***A1*** denotes 10 μm. Colorbars indicate the range of ΔF/F values in percent. ***D***, Mean percent of the AL activated by the odor, ±SEM, as determined by the number of pixels above an intensity equal to the 40th percentile of the dynamic range of all AL pixels for a given fly. Samples of this region are outlined in white dashes in ***A4***, ***B4***, and ***C4***. Dashed lines denote statistical significance in the difference between the indicated R32F10-Gal4 odor concentration pairs (ANOVA, *p* = 0.00005, with Tukey’s *post hoc* test, *p* values: 10^−10^ and 10^−8^ = 0.9677; 10^−10^ and 10^−6^ = 0.0379; 10^−10^ and 10^−4^ = 0.0013; 10^−8^ and 10^−6^ = 0.1047; 10^−8^ and 10^−4^ = 0.0049; 10^−6^ and 10^−4^ = 0.5954). Concentration pairs for R70A09-Gal4 and NP3056-Gal4 were not significant (ANOVA, *p* = 0.6383 and 0.3374, respectively). *n* = 11, 12, and 10 for NP3056-Gal4, R70A09-Gal4, and R32F10-Gal4, respectively.

Stronger concentrations of an odor activate more ORs (Hallem and Carlson, 2006) and because ORNs which express the same OR all project to the same glomerulus, the number of glomeruli activated by an odor increases with concentration (Hallem and Carlson, 2006). Previous literature has demonstrated that spiking LN populations do not change activation patterns across concentrations; they only change in intensity (Hong and Wilson, 2015). This indicates that spiking LNs propagate their signals across the AL regardless of the number of glomeruli activated. We sought to determine whether there is glomerular specificity in the nonspiking LNs by calcium imaging during odor exposures of increasing concentration. We hypothesized that if nonspiking LN responses are restricted to active glomeruli, increased odor concentrations will activate more glomeruli and therefore increase the nonspiking LN activity across the space of the AL. For NP3056-Gal4 and R70A09-Gal4, we observed a similar result to previous literature; the activation pattern in the focal plane did not vary across concentrations (Fig. 6*A*,*B*; Hong and Wilson, 2015). However, in R32F10-Gal4, we observed regions of activation which increased in size with increasing odor concentration (Fig. 6*C*). The differences in the percent of the imaging plane activated by odor concentrations in R32F10-Gal4 displayed an upward trend (Fig. 6*D*). Differences were significant between odor concentrations at dilutions separated by at least 10^−4^ (Fig. 6*D*). However, within spiking lines, varying odor concentrations produced neither a consistent directional trend nor statistically significant differences within the lines (Fig. 6*D*).

## Discussion

In this work, we report the observation of nonspiking patchy LNs in the *Drosophila* AL. While not uncommon in other insects, nonspiking LNs were not previously described in the early olfactory circuitry of the fly. We have demonstrated their unique nonspiking characteristic through electrophysiology, immunohistochemistry, and *in situ* hybridization. Further, we have investigated the potential physiology of these neurons through calcium imaging during odor responses.

### Common to other insects, organisms, and sensory systems, nonspiking LNs are a novel observation in the adult *Drosophila* AL

While neurons which fire action potentials are considered the norm, nonspiking olfactory interneurons are present across many insect species (Laurent and Davidowitz, 1994; Husch et al., 2009; Tabuchi et al., 2015). In *Drosophila*, it was previously assumed the entire population of LNs in the adult AL were spiking (Seki et al., 2010; Wilson et al., 2004). Here, we present a population of adult *Drosophila* AL LNs which fail to fire action potentials. This contrasts with other insects, such as the locust in which nonspiking cells are the prevalent olfactory LN type (Laurent and Davidowitz, 1994). Other species such as the cockroach are known to have both spiking and nonspiking LNs in the AL (Husch et al., 2009). Early in development, spiking neurons are not common in *Drosophila* as most neurons of the larval brain are *para* negative (Ravenscroft et al., 2020). Other neurons in the adult *Drosophila* brain have also been identified as nonspiking. For instance, a variety of neurons, including interneurons, in the fly visual system are nonspiking (Behnia et al., 2014; Gruntman et al., 2021; Kohn et al., 2021). The lack of action potentials in these neurons may facilitate local processing and allow fewer neurons in the invertebrate CNS to perform the role of multiple cell classes in animals with a greater number of neurons. For instance, in the mushroom body (MB), a pair of neurons known as the anterior paired lateral neurons (APLns) are the fly analogs to the locust’s nonspiking giant GABAergic neurons (Leitch and Laurent, 1996), and are also believed to be nonspiking (X. Liu and Davis, 2009). Each APLn provides localized inhibition to Kenyon cells of the MB, despite innervating the entire region (Tanaka et al., 2008). The APLn’s activity is spatially restricted (Inada et al., 2017; Amin et al., 2020) and its *para* transcript level is low (Amin et al., 2020), supporting the hypothesis that this pair of cells is nonspiking and local processing.

Nonspiking cells are also common in noninsect organisms. For example, several amacrine cells of the vertebrate retina are nonspiking (Gleason et al., 1993). Like olfactory LNs, these cells are often GABAergic, axonless, and observed in a variety of morphologic patterns. While most *Drosophila* neurons only start spiking in the adult, some amacrine cells start as spiking cells during development then lose spiking when the retina reaches maturity (Zhou and Fain, 1996). They are believed to have roles in center-surround responses (Bloomfield, 1992) as well as contrast adaptation (Dunn et al., 2006), and the nonspiking characteristic is thought to contribute to localized synaptic release without propagation across the whole cell (Grimes et al., 2010).

### Nonspiking LNs lack translation of Para channels, thereby preventing sodium current-dependent action potentials

Patch-clamp electrophysiology revealed these cells lack detectable voltage-gated sodium current, and immunohistochemistry showed this is because of a downregulation of the voltage-gated sodium channel Para. Under strong odor stimulation and current injection, we did not observe action potentials in R32F10-Gal4 LNs. This was true for multiple recordings within flies, across flies, and between the Gal4 and LexA versions of the R32F10 promoter fragment driver. Taken together, these data confirm that our observation of nonspiking cells was not an artifact of our recording setup or technique but rather a reproducible result.

*Drosophila* express a single voltage-gated sodium channel, Para (Germeraad et al., 1992), which is blocked by TTX (Warmke et al., 1997). We did not observe TTX-sensitive sodium current in voltage-clamp recordings of nonspiking LNs. However, it is possible this result could be attributed to poor space clamp: an electrophysiological recording issue in which the control of the cell’s membrane potential diminishes with distance from the electrode. *Drosophila* LNs are particularly susceptible to poor space clamp as their processes are small and spike initiation sites are often located far from the soma. Despite this, we were consistently able to see TTX-sensitive currents from spiking cells which have similar physical attributes to nonspiking LNs despite poor space clamp, and therefore we attribute our result to the physiology of the cells and not the recording technique.

Consistent with the nonspiking phenotype, we also used the FlpTag technique (Fendl et al., 2020) to demonstrate the lack of *para* expression in R32F10-Gal4 patchy LNs. The results from this experiment further validate the absence of sodium current. Para has been previously shown to localize in distal axon segments which resemble mammalian action potential initiation sites (Ravenscroft et al., 2020), and our results for R70A09-Gal4 spiking local interneurons are consistent with this finding with most of the staining located in the primary neurites. In contrast, we observe significantly less Para-FlpTag signal in the ALs of R32F10-Gal4 flies, indicative of the labeled LNs lacking expression. Furthermore, neurons outside of the AL labeled by R32F10-Gal4 still stain positively for Para, showing the lack of signal within this driver line is unique to the nonspiking patchy AL LNs. Because this technique is dependent on Flp activity driven by binary expression, it is subject to developmental timing of expression and stochasticity. As such, we observed stochasticity between hemispheres of most brains labeled by FlpTag. Often, this manifested in one AL receiving denser labeling than the other. Despite the stochastic labeling, we still observed significant differences in the Para signal intensity. The downregulation of *para* translation may be a common theme in nonspiking neurons in *Drosophila*. Similar to the R32F10-Gal4 LNs, multiple classes of nonspiking cells in the *Drosophila* visual system continue to express *para* transcript but fail to generate typical sodium action potentials (Davis et al., 2020).

Although R32F10-Gal4 LNs lack sodium channels and current, they still produce *para* transcript. It is important to note transcription and translation are not necessarily correlated. In developing *Drosophila*, it has been shown that genes which have downregulated transcripts may have upregulated protein products and vice versa (Becker et al., 2018). Therefore, we surmise *para* expression in these cells is subject to post-transcriptional regulation. In larval flies, translational repressor Pumilio works with Nanos and Brain Tumor to regulate *para* transcripts in motoneurons (Muraro et al., 2008), and we suspect a similar mechanism prevents translation in the adult nonspiking LNs, perhaps even using the same repressors. Alternatively, it is possible these cells are nonspiking until a certain critical period or event, at which point sodium channels may be produced. This could be a mechanism for localized plasticity, where the patchy morphology could work with selective expression to achieve glomerular specific modulation.

### Odor responses of nonspiking LNs allude to potential roles in olfactory coding and AL activity regulation

Nonspiking LNs could serve a variety of functions in the *Drosophila* AL. In addition to their nonspiking characteristic, this population of LNs has the patchy morphologic pattern. This morphology is distinct from other anatomic patterns of LNs, as the processes innervating a given glomerulus tend to be isolated from the neurites in other glomeruli (Chou et al., 2010). Furthermore, the vast majority of LNs are GABAergic, and other Gal4 lines which label patchy cells are predominantly GABA-positive (Chou et al., 2010). From our calcium imaging experiments, we suspect the glomerular compartments of these cells to be electrotonically isolated, such that activation within one glomerulus would not propagate broadly across the cell. This is evidenced by the distinct patterns of activation unique to each odor, the increasing size of the active region with increasing odor concentration, and the regionally isolated spontaneous activity. The exception to this finding was the correlation in response pattern for pentyl acetate and valeric acid for nonspiking LNs. We speculate the high correlation is because of the number of shared glomeruli in the focal plane activated by both odors (Hallem and Carlson, 2006; Silbering et al., 2011).

One potential role for nonspiking LNs is intraglomerular inhibition (Fusca and Kloppenburg, 2021). This is a phenomenon in which the cognate glomerulus for a given odor is subject to extra inhibition compared with the other glomeruli (Root et al., 2011; Hong and Wilson, 2015). It is unclear which LNs are the source of this boost of inhibition, and the matter is further complicated if all LNs propagate activity across their processes through spiking. One potential answer to this problem is uniglomerular LNs. In the mouse olfactory bulb, glomeruli have locally-projecting periglomerular cells. While their role is not fully understood, periglomerular cells are known to receive both feedforward and feedback inputs and they serve as a source of local presynaptic and postsynaptic inhibition (Aungst et al., 2003; Murphy et al., 2005; Wachowiak et al., 2005). Periglomerular cells innervate a single glomerulus but most *Drosophila* LNs innervate many glomeruli (Chou et al., 2010) and therefore it is not likely that intraglomerular inhibition is because of uniglomerular LNs. However, a nonspiking neuron with electronically isolated compartments could theoretically emulate a network of uniglomerular neurons to achieve region-specific inhibition.

During odor responses, the population of nonspiking LNs showed patterns of activation which did not correlate between odors. This contrasts with spiking LNs, which have been shown both in this work and in previous literature to have pan-glomerular, odor-identity independent activation during responses (Fusca and Kloppenburg, 2021; Hong and Wilson, 2015). This wide-spread activation causes lateral inhibition and enhances odor discriminability through divisive gain control (Olsen and Wilson, 2008; Root et al., 2008; Olsen et al., 2010; Oizumi et al., 2012). Strong odor responses are inhibited more than weak responses, functionally amplifying weak inputs and preventing saturation from strong inputs. This results in AL neurons using their full dynamic range while simultaneously increasing odor contrast. The differential activation patterns of nonspiking LNs could serve to provide local inhibition alongside the lateral inhibition of spiking LNs to further prevent the most active glomeruli from reaching saturation.

Increasing concentrations of odorants activate more ORNs (Hallem and Carlson, 2006). We observed an increase in the activated area of nonspiking LNs as odor concentration increased. This stands to support the hypothesis that these LNs are spatially isolated in their activity; ORNs signaling to more glomeruli would activate more nonspiking LN compartments. This spatial isolation could contribute to noise regulation in the AL. PNs are innately noisy because of a resting potential near threshold, and a response is triggered when ORN spike times are correlated, even if coincidently (Franks, 2015; Jeanne and Wilson, 2015). A strictly global model of inhibition would theoretically regulate the noise, but the widespread inhibition would also decrease sensitivity. Local intraglomerular inhibition from a compartmentalized nonspiking neuron could spatially inhibit noise arising from spontaneous activity, therefore preventing false positive responses and/or limiting the duration of bursts in PNs while maintaining sensitivity in other glomeruli. Additionally, intraglomerular inhibition could aid in preventing odor response saturation, thus enhancing odor coding across concentrations. We posit this as a potential role for nonspiking LNs in the *Drosophila* AL.

## Synthesis

Reviewing Editor: Miriam Goodman, Stanford University Decisions are customarily a result of the Reviewing Editor and the peer reviewers coming together and discussing their recommendations until a consensus is reached. When revisions are invited, a fact-based synthesis statement explaining their decision and outlining what is needed to prepare a revision will be listed below. The following reviewer(s) agreed to reveal their identity: Marion Silies, Yu-Chieh Chen.

The authors use electrophysiology, calcium imaging, and immunofluorescence imagining to examine one population of non-spiking local interneurons (LNs) in the antennal lobe (AL) of Drosophila olfactory system.

To genetically label distinct population of local interneurons, the authors three enhancer-GAL4 lines to (R70A09-GAL4, R32F10-GAL4, and NP2056-GAL4). Making using of these labels, they characterized the morphological and electrical properties of these local interneurons. They found that the R70A09-GAL4 neurons exhibit pan-neuron AL innervation and showed clear voltage-gated Na+ currents and generated action potentials that can be blocked by TTX. In contrast, the R32F10-GAL4 neurons exhibit patchy AL innervation and showed graded potentials with no voltage-gated Na+ current. They used HCR in situ hybridization to show that para, the only gene encodes a voltage-gated Na+ channel in flies was expressed in both spiking and non-spiking LNs. With Flptag technique, the authors found that the para-GAP protein showed little if any expression in non-spiking LNs, suggesting a post-transcriptional downregulation of para in these non-spiking LNs. Lastly, they use functional calcium imaging to probe the potential function of this unique non-spiking LNs in odor coding. They found that the non-spiking LNs showed more variable spatial patterns in response to different odors. The author speculated that the unique property of non-spiking LNs might shape odor coding via intraglomerular inhibition.

In general, this characterization of non-spiking LNs in Drosophila olfactory system will be important for further characterize the functional roles of distinct LNs in odor coding. The findings presented in this manuscript present a nice starting point for a better characterization of non-spiking LNs.

Rev1 notes two kinds of general concerns, one is that some claims are overstated and the second is that calcium imaging via wide-field imaging may limit conclusions regarding LN function and signaling (see below for specific examples of these limitations and suggestions for addressing them). Rev2 provides overlapping feedback, with additional suggestions for improvement. Collectively, their feedback suggests improvements to the text, streamlining data presentation, and proposes that additional experiments to solidify the morphological characterization of ‘patchy’ LNs and those that appear to generate pan-neuronal innervation of the AL would increase the rigor of the study and confidence in the classification of these types of LNs.

Reviewer 1

Major critiques

• The manuscript oversells several claims that are made, including:

(1) For example, when reading the introduction or the abstract, the non-expert author is led to believe that the discovery of nonspiking LNs is novel, whereas these have been described in many species, including moths and cockroaches (Tabuchi et al. 2015, Fuscan and Kloppenburg, 2020). Furthermore, it was known that (spiking) LNs can be physiologically distinct (e.g. Seki et al. 2010). While all of these papers are mentioned in the discussion, they should be essentials parts of an introduction that better reflects the current state of knowledge.

(2) I am convinced by the finding that these are non-spiking LNs, but I am surprised that the authors did not consider the possibility that Para can also have a role in non-spiking neurons. There are actually many non-spiking neurons, for example in the Drosophila visual system, that express Para (Davis et al. 2020). Can this possibility at least be discussed? Especially after seeing para transcript in LNs, I no longer “expected” (P9, bottom) to see less Para protein in non-spiking LNs. [editor’s note: In principle, a neuron that expresses voltage-gated Na+ channels at low density might well fail to generate action potentials.]

(3) The authors should discuss the limitations of the recording techniques, especially wide-field imaging of calcium signals.

• Figure 1: According to the figure legend, the projections of the LNs in these two lines are shown with very different techniques: stochastic labeling via SPARC, or cell fills. To convincingly show that one LN type is patchy, whereas the other isn’t, this must be done with the same technique. The full expression pattern of the lines used in this study should be shown here, or somewhere in the manuscript, e.g. in Figure 3.

• Figure 2 / P9: A negative (e.g. antisense) control is missing for the HCR.

The term “The promotor line” should be replaced with “driver line” throughout the manuscript. Two of the driver lines used in this study are in fact promotor-Gal4 fusions, but the NP line is an enhancer trap.

• P9: “Para localizes to active zones of neurons”: This clearly cannot be claimed from images at this resolution. LNs have both pre- and postsynaptic sites in this region, and even this cannot be distinguished based on these images. It would also help to see the localization results more clearly if the GFP channel was also shown alone.

• Some figures contain much information that directly relates to the content of other figures, making 8 full figures a bit unnecessary. I would suggest to combine Figure 3 and 4, as well as 5 and 6 (or even 5-7), and I do not think that the presence of Figure 8 adds much to this manuscript.

• Figure 4 / P10:

Figure 4 is just showing snapshots of an individual brains. This figure should at least show time traces, of several flies, to be informative, and also show temporal aspects of the odor response. The description of Fig. 4 results (P10) could be improved: which ones are the public and the private odors? Which concentration was used?

• P10 bottom: I wasn’t convinced when I first read the conclusions that patch 32F10-Gal4 cells displayed spatially variable patterns of activation. To me, it sounded like I was comparing the activity to the ones of the other LN lines. The main point, that could be made clearer, is that different odors elicited responses in different regions, in the same brain/fly. Furthermore, is it possible to comment on glomeruli identities here, or is this impossible with wide field imaging techniques?

• P12: I am not convinced that the authors can really exclude the possibility that higher concentrations mostly lead to the recruitment of glomeruli who are below detection threshold at higher concentrations. This is supported by the fact that wide field imaging was used here

Minor comments:

1. Abstract: to determine *if* their nonspiking characteristics

2. P4: mCD8::GFP, not mDC8

3. P5: The material & methods section is cryptic in several places, or involves lab slang, and could benefit from proof reading by another expert (maybe a native speaker). For example, what does “current signals were conditioned using positive/negative subtraction” mean? “active components” are action potentials?

4. P6: what microscope was used for wide field imaging?

5. "Changes in fluorescence were calculated at ... normalized to baseline (dF/F)”.

dF/F is not baseline. This should read “changes in fluorescence (dF/F) were calculated as (fix typo here)...

6. P10: “To assess the physiological role” - The physiology of these neurons was assessed, but not their role.

7. The term “The promotor line” should be replaced with “driver line” throughout the manuscript. Two of the driver lines used in this study are promotor-Gal4 fusions, but the NP line is an enhancer trap, not a promotor fusion.

8. P10: “R70A09-Gal4 labels a similar number of LNS...” - this is described twice, here and on page 8.

9. P10: “As shown in previous literature...” - citations are missing in this sentence

10. P3: You did not demonstrate that “these LNs are unique to Drosophila”. Non-spiking LNs exist in other species, and the LNs studied are also just one class, and are likely not the only non-spiking LNs in Drosophila.

11. P11: “and pooled results which were comparable between flies”. - This sounds like the authors cherry picked only pooled results that were comparable . I hope that there is just a comma missing and that this should read “and pooled results, which were comparable between flies"

12. P15: “we observe significantly less Para singals in the ALs of R32F10-Gal4 flies” - This is not a true statement. You observe less Para in cells marked by R32F10-Gal4 as compared to the other LNs you are looking at, but not in the ALs of these flies overall.

13. P15: “This results seems contrary to the other data” - I do not see the contradiction. There is mRNA, but less protein, and the neurons are non-spiking. Post-transcriptional regulation seems like a perfectly non-controversial mechanisms to achieve this.

14. Figure 1 caption: “Other odors tested included...”. - Is this information relevant to this figure? It does not seem to be. Also, the panel calls are wrong towards the end of the figure caption: G) should be H), H) should be I) ?

15. Figure 2 legend: What is marked in red / blue / white in D/E?

Reviewer 2

Rev2 noted some minor critiques and suggestions for improvement. Many related to the presentation of experimental data, including some that overlap with Rev1’s comments.

1. The authors mentioned in the text many times that the number of cells labeled by the R70A09-GAL4 and R32F10-GAL4 are similar. They further showed the quantified numbers of cells labeled in each line in Fig. 3C and D (11.5 neurons in R70A09-GAL4; 12.8 neurons in R32F10-GAL4). However, the expression images shown in Fig. 1A and B showed that the non-spiking LNs had a much restrictive patchy expression while spiking LNs innervate more or less pan-neuronally. Do the images in Fig. 1A and B only label 1 neuron? Can the author provide a better morphological description of a single non-spiking LN and spiking LN? Having a cartoon diagram showing their morphological difference would help explaining their potential functions. Performing single cell labeling with MCFO (multip color flip out) would help showing the morphological features of non-spiking LN and spiking LN neuron.

2. Page 21, Fig1 legend. The G) and H) should be H) and I). They were mis-labeled.

3. Page 15, discussion, the authors said “Furthermore, neurons outside of the AL labeled by R32F10-Gal4 still stain positively for Para, showing the lack of signal is unique to the nonspiking patchy AL LNs.” I think this is an important point and should be moved to the result section. In addition, the positive para signals outside of the AL should be indicated in the figure as well.

4. In Fig. 2C, the “sparse” on the X axis label is confusing here. I understand it means “sparse” neurons as compared to “dense” labeled neurons by NP3056-GAL4. However, you did not explain what “sparse” means here and wasn’t until the Fig. 3 that I realized what you are referring to. Please either clarify the terminology earlier or remove the “sparse” in Fig. 2.

5. What’s the source of the Ca in non-spiking LNs while they don’t fire action potentials?

6. Fig. 8 is not adding too much to the story and is very confusing. The graph is not easy to understood. Consider remove it and mention the observation of spontaneous calcium response in the text.

7. In all the Ca imaging results, the authors used the peak response to determine the spatial variability in non-spiking LNs. What’s missing is the temporal axis? Is there anything unique in terms of the temporal responses? Could the authors perform analyses and the plots for the temporal Ca responses between non-spiking LNs and spiking LNs?

## Author Response

Synthesis of Reviews:

Synthesis Statement for Author (Required):

The authors use electrophysiology, calcium imaging, and immunofluorescence imagining to examine one population of non-spiking local interneurons (LNs) in the antennal lobe (AL) of Drosophila olfactory system.

To genetically label distinct population of local interneurons, the authors three enhancer-GAL4 lines to (R70A09-GAL4, R32F10-GAL4, and NP2056-GAL4). Making using of these labels, they characterized the morphological and electrical properties of these local interneurons. They found that the R70A09-GAL4 neurons exhibit pan-neuron AL innervation and showed clear voltage-gated Na+ currents and generated action potentials that can be blocked by TTX. In contrast, the R32F10-GAL4 neurons exhibit patchy AL innervation and showed graded potentials with no voltage-gated Na+ current. They used HCR in situ hybridization to show that para, the only gene encodes a voltage-gated Na+ channel in flies was expressed in both spiking and non-spiking LNs. With Flptag technique, the authors found that the para-GAP protein showed little if any expression in non-spiking LNs, suggesting a post-transcriptional downregulation of para in these non-spiking LNs. Lastly, they use functional calcium imaging to probe the potential function of this unique non-spiking LNs in odor coding. They found that the non-spiking LNs showed more variable spatial patterns in response to different odors. The author speculated that the unique property of non-spiking LNs might shape odor coding via intraglomerular inhibition.

In general, this characterization of non-spiking LNs in Drosophila olfactory system will be important for further characterize the functional roles of distinct LNs in odor coding. The findings presented in this manuscript present a nice starting point for a better characterization of non-spiking LNs.

Rev1 notes two kinds of general concerns, one is that some claims are overstated and the second is that calcium imaging via wide-field imaging may limit conclusions regarding LN function and signaling (see below for specific examples of these limitations and suggestions for addressing them). Rev2 provides overlapping feedback, with additional suggestions for improvement. Collectively, their feedback suggests improvements to the text, streamlining data presentation, and proposes that additional experiments to solidify the morphological characterization of ‘patchy’ LNs and those that appear to generate pan-neuronal innervation of the AL would increase the rigor of the study and confidence in the classification of these types of LNs.

Reviewer 1

Major critiques

• The manuscript oversells several claims that are made, including:

(1) For example, when reading the introduction or the abstract, the non-expert author is led to believe that the discovery of nonspiking LNs is novel, whereas these have been described in many species, including moths and cockroaches (Tabuchi et al. 2015, Fuscan and Kloppenburg, 2020). Furthermore, it was known that (spiking) LNs can be physiologically distinct (e.g. Seki et al. 2010). While all of these papers are mentioned in the discussion, they should be essentials parts of an introduction that better reflects the current state of knowledge.

We thank the reviewer for this suggestion. We have revised the abstract and introduction accordingly and stressed that non-spiking LNs have been previously reported in the antennal lobe of other insects (Husch et al., 2009; Laurent and Davidowitz, 1994; Tabuchi et al., 2015). We also report that non-spiking LNs have been found elsewhere in the Drosophila CNS.

(2) I am convinced by the finding that these are non-spiking LNs, but I am surprised that the authors did not consider the possibility that Para can also have a role in non-spiking neurons. There are actually many non-spiking neurons, for example in the Drosophila visual system, that express Para (Davis et al. 2020). Can this possibility at least be discussed? I just could not really find this anywhere in Davis et al 2020.

We have now amended the discussion section accordingly. We reference that neurons in the visual system do express the para mRNA despite being nonspiking. We now emphasize that this might be a theme that is common in nonspiking neurons in Drosophila and that nonspiking neurons may support local computations.

Especially after seeing para transcript in LNs, I no longer “expected” (P9, bottom) to see less Para protein in non-spiking LNs. [editor’s note: In principle, a neuron that expresses voltage-gated Na+ channels at low density might well fail to generate action potentials.]

We have revised this portion of the results and removed the statement that the lack of Para was surprising. It is possible that these LNs express Para in very low levels that are below our detection threshold. We tried to alleviate this concern by using multiple strategies (electrophysiology and FlpTag).

(3) The authors should discuss the limitations of the recording techniques, especially wide-field imaging of calcium signals.

We now discuss the limitations of patch clamp electrophysiology (space clamp issues) and the limitations of wide-field imaging. We mention why we believe these limitations do not negate our findings. Specifically, space clamp issues associated with physiology are complimented by the FlpTag approach which also suggest little to no Para expression. Our FlpTag experiments also benefitted from the amplification of signal inherent to immunochemistry protocols. Thus, the technique should be quite sensitive at detecting Para protein.

Wide-field imaging suffers from contamination of signals above and below the focal plane. We now acknowledge this limitation. However, even given this limitation, it is difficult to explain how signals in spiking LN lines (NP3056 and R70A09) show similar spatial patterns of activation across odor types yet R32F10 shows spatially distinct responses. Previous studies using 2-photon calcium imaging have also shown spatially uniform odor responses in NP3056 (cited in the text as Hong et al. 2015), consistent with our results. We suspect that large signals originating from outside the focal plane in R32F10 would be likely to make the odor responses broader and thus resemble the responses of spiking LN lines more. Thus, we argue that the limitations of wide-field imaging are more likely to underestimate the size of our results rather than exaggerate them.

• Figure 1: According to the figure legend, the projections of the LNs in these two lines are shown with very different techniques: stochastic labeling via SPARC, or cell fills. To convincingly show that one LN type is patchy, whereas the other isn’t, this must be done with the same technique. The full expression pattern of the lines used in this study should be shown here, or somewhere in the manuscript, e.g. in Figure 3.

We now provide SPARC images for both the pan-glomerular and patchy LNs in Figure 1. We have also included images of the full expression patterns of each driver line in Supplementary Figure 1. R70A09 and R32F010 full images can also be seen on FlyLight from Janelia Farms.

• Figure 2 / P9: A negative (e.g. antisense) control is missing for the HCR.

We now include a new supplementary figure, Fig. S2, which includes a negative control. The larval brain is known to be para-negative while the larval nerve cord does express para. By staining larval CNS we can see transcription in the nerve cord and report its absence in the brain. This is consistent with previous reports (Ravencroft et al. 2020) and demonstrates that our reagent works as expected.

The term “The promotor line” should be replaced with “driver line” throughout the manuscript. Two of the driver lines used in this study are in fact promotor-Gal4 fusions, but the NP line is an enhancer trap.

This has been corrected in the manuscript.

• P9: “Para localizes to active zones of neurons”: This clearly cannot be claimed from images at this resolution. LNs have both pre- and postsynaptic sites in this region, and even this cannot be distinguished based on these images. It would also help to see the localization results more clearly if the GFP channel was also shown alone.

We apologize for the confusion. We intended to state that Para localized to the spike initiation zone. This region was referred to as the distal axon segment (DIS) in Ravencroft et al. 2020. We have replaced active zone to action potential initiation site and cited Ravencroft et al. 2020.

• Some figures contain much information that directly relates to the content of other figures, making 8 full figures a bit unnecessary. I would suggest to combine Figure 3 and 4, as well as 5 and 6 (or even 5-7), and I do not think that the presence of Figure 8 adds much to this manuscript.

We have now consolidated a few of the figures, but also added raw ΔF/F traces and basic statistics of the response’s temporal features. We have also taken the reviewer’s suggestion and removed Figure 8 from the manuscript and all discussion of spontaneous activity recordings.

• Figure 4 / P10:

Figure 4 is just showing snapshots of an individual brains. This figure should at least show time traces, of several flies, to be informative, and also show temporal aspects of the odor response. The description of Fig. 4 results (P10) could be improved: which ones are the public and the private odors? Which concentration was used?

We have revised Figure 4 and now include the ΔF/F traces so that the reader can see the temporal properties of the responses. We now include basic statistics on the latency, time to half-max, decay time, and response duration. We have rephrased our descriptions to be more accessible to the common reader and state concentrations used.

• P10 bottom: I wasn’t convinced when I first read the conclusions that patch 32F10-Gal4 cells displayed spatially variable patterns of activation. To me, it sounded like I was comparing the activity to the ones of the other LN lines. The main point, that could be made clearer, is that different odors elicited responses in different regions, in the same brain/fly. Furthermore, is it possible to comment on glomeruli identities here, or is this impossible with wide field imaging techniques?

The reviewer has it correct, and we tried to make this clearer in the manuscript now. We revised the manuscript to emphasis our analyses are aimed at differences between odors within individual flies. While we have a lot of confidence in the location of various glomeruli, (such as the DA1 glomerulus that responds to cVA), this would be too difficult a claim to make given our data. This is less because of the wide-field imaging approach, but more a limitation of the LN projection patterns which do not clearly demarcate glomeruli. Determining glomeruli identity is easily possible with wide-field PN or ORN recordings, but much more difficult here.

• P12: I am not convinced that the authors can really exclude the possibility that higher concentrations mostly lead to the recruitment of glomeruli who are below detection threshold at higher concentrations. This is supported by the fact that wide field imaging was used here. We do not exclude this possibility.

We believe that two factors almost certainly contribute to the broadening of R32F10 LN odor responses with increasing odor concentration. It is well known from the previous literature that increasing odor concentration increases the ORN classes and thus glomeruli that are recruited (Hallem and Carlson 2006). R32F10 LNs (as a population) also have postsynaptic specializations throughout the entire antennal lobe (Sizemore et al. 2022). Thus, it is likely that higher odor concentrations activate new glomeruli and inputs onto R32F10 LNs. However, we also agree with the reviewer that there must be some signals that are simply below our detection threshold at low concentrations and suddenly above threshold with increasing concentration. More importantly, this behavior has not been reported in broad spiking LN lines by others (Hong and Wilson 2015 using 2-photon) and we did not observe it. Generally, even the weakest odor concentrations continue to give an antennal lobe-wide response in spiking LN lines.

Minor comments:

1. Abstract: to determine *if* their nonspiking characteristics

Thank you, we’ve now corrected this.

2. P4: mCD8::GFP, not mDC8

Thank you, we’ve now corrected this.

3. P5: The material & methods section is cryptic in several places, or involves lab slang, and could benefit from proof reading by another expert (maybe a native speaker). For example, what does “current signals were conditioned using positive/negative subtraction” mean? “active components” are action potentials?

Thank you, we’ve now expanded the methods section to clarify the positive/negative subtraction protocol. Active components of the voltage clamp traces are generally voltage sensitive conductances rather than passive properties. We’ve revised this section to make this clearer now.

4. P6: what microscope was used for wide field imaging?

This is now included in the methods.

5. “Changes in fluorescence were calculated at ... normalized to baseline (dF/F)”.

dF/F is not baseline. This should read “changes in fluorescence (dF/F) were calculated as (fix typo here)...

Thank you, we’ve now corrected this.

6. P10: “To assess the physiological role” - The physiology of these neurons was assessed, but not their role.

Thank you, we’ve now changed physiological role to physiology.

7. The term “The promotor line” should be replaced with “driver line” throughout the manuscript. Two of the driver lines used in this study are promotor-Gal4 fusions, but the NP line is an enhancer trap, not a promotor fusion.

Thank you, we’ve now corrected this.

8. P10: “R70A09-Gal4 labels a similar number of LNS...” - this is described twice, here and on page 8.

Thank you, we’ve now corrected this by removing this description from page 8.

9. P10: “As shown in previous literature...” - citations are missing in this sentence

As shown in previous literature

Thank you, we’ve now added references to support these claims.

10. P3: You did not demonstrate that “these LNs are unique to Drosophila”. Non-spiking LNs exist in other species, and the LNs studied are also just one class, and are likely not the only non-spiking LNs in Drosophila.

Yes, we stress that non-spiking LNs have been observed in other insects and that other non-spiking neurons are also present in Drosophila.

11. P11: “and pooled results which were comparable between flies”. - This sounds like the authors cherry picked only pooled results that were comparable . I hope that there is just a comma missing and that this should read “and pooled results, which were comparable between flies"

We thank the reviewer for pointing this out and we have revised that portion of the results.

12. P15: “we observe significantly less Para singals in the ALs of R32F10-Gal4 flies” - This is not a true statement. You observe less Para in cells marked by R32F10-Gal4 as compared to the other LNs you are looking at, but not in the ALs of these flies overall.

Thank you. We have revised this to now read less Para-FlpTag signal, which should be more accurate than our previous statement.

13. P15: “This results seems contrary to the other data” - I do not see the contradiction. There is mRNA, but less protein, and the neurons are non-spiking. Post-transcriptional regulation seems like a perfectly non-controversial mechanisms to achieve this.

We have revised this statement accordingly in the manuscript.

14. Figure 1 caption: “Other odors tested included...”. - Is this information relevant to this figure? It does not seem to be. Also, the panel calls are wrong towards the end of the figure caption: G) should be H), H) should be I) ?

We ultimately wanted to convey to the reader that we tested a broader number of odors, and that spiking was still never observed. We are confident that the non-spiking nature of these cells is not due to a lack of finding the optimal stimulus, but rather that it is a feature of the neurons and their lack of Para expression. We have fixed the figure legend call outs.

15. Figure 2 legend: What is marked in red / blue / white in D/E?

We have now specified the colors in this and other figures.

Reviewer 2

Rev2 noted some minor critiques and suggestions for improvement. Many related to the presentation of experimental data, including some that overlap with Rev1’s comments.

1. The authors mentioned in the text many times that the number of cells labeled by the R70A09-GAL4 and R32F10-GAL4 are similar. They further showed the quantified numbers of cells labeled in each line in Fig. 3C and D (11.5 neurons in R70A09-GAL4; 12.8 neurons in R32F10-GAL4). However, the expression images shown in Fig. 1A and B showed that the non-spiking LNs had a much restrictive patchy expression while spiking LNs innervate more or less pan-neuronally. Do the images in Fig. 1A and B only label 1 neuron? Can the author provide a better morphological description of a single non-spiking LN and spiking LN? Having a cartoon diagram showing their morphological difference would help explaining their potential functions. Performing single cell labeling with MCFO (multip color flip out) would help showing the morphological features of non-spiking LN and spiking LN neuron.

We now provide a SPARC image for both a spiking and a non-spiking LN in figure 1. Our collaborator, Dr. Andrew Dacks at West Virginia University, has extensively examined the anatomy and morphology of the R32F10 patch LNs in his latest manuscript available on BioArxiv (https://www.biorxiv.org/content/10.1101/2022.04.27.489804v2). As we knew his team was handling the morphology and connectivity of these neurons, we focused on their physiology and non-spiking attribute.

2. Page 21, Fig1 legend. The G) and H) should be H) and I). They were mis-labeled.

Thank you, we have now revised this.

3. Page 15, discussion, the authors said “Furthermore, neurons outside of the AL labeled by R32F10-Gal4 still stain positively for Para, showing the lack of signal is unique to the nonspiking patchy AL LNs.” I think this is an important point and should be moved to the result section. In addition, the positive para signals outside of the AL should be indicated in the figure as well.

Thank you for the suggestion. We have revised the section accordingly.

4. In Fig. 2C, the “sparse” on the X axis label is confusing here. I understand it means “sparse” neurons as compared to “dense” labeled neurons by NP3056-GAL4. However, you did not explain what “sparse” means here and wasn’t until the Fig. 3 that I realized what you are referring to. Please either clarify the terminology earlier or remove the “sparse” in Fig. 2.

We have removed mentions of “sparse” and “dense,” as upon review we decided they did not add to the manuscript.

5. What’s the source of the Ca in non-spiking LNs while they don’t fire action potentials?

This is a very interesting question that we unfortunately do not know the answer to. We are simply using calcium imaging as a proxy for activation. The Drosophila genome encodes multiple voltage-gated calcium channels. Additional Ca++ signal could also result from calcium-induced calcium release from internal stores. Unfortunately, we did not have time to investigate this in our study.

6. Fig. 8 is not adding too much to the story and is very confusing. The graph is not easy to understood. Consider remove it and mention the observation of spontaneous calcium response in the text.

We have removed the figure per both reviewers’ request and now discuss spontaneous activity briefly in the discussion.

7. In all the Ca imaging results, the authors used the peak response to determine the spatial variability in non-spiking LNs. What’s missing is the temporal axis? Is there anything unique in terms of the temporal responses? Could the authors perform analyses and the plots for the temporal Ca responses between non-spiking LNs and spiking LNs?

We now include sample traces of the deltaF/F responses so the reader can appreciate the temporal qualities of the responses. We performed statistics on the basic properties of the responses, but we did not observe any obvious differences between spiking and non-spiking LNs that would give us any additional insight into their function. We now present these temporal data in Figure 4.
